# Supplementary material for: Web-Based Assessment of the Phenomenology of Autobiographical Memories in Young and Older Adults
Source: Brain Sci. 2021 May 18;11(5):660. doi: 10.3390/brainsci11050660 (PMC8158337; doi:10.3390/brainsci11050660)
Supplement: Supplementary file 1 [file brainsci-11-00660-s001.zip › brainsci-1178399-supplementary.pdf]

R packages used in this work (alphabetical order)

Package	Version	Citation	Analysis
boot	1.3.20	Canty, A.; Ripley, B. boot: Bootstrap R (S-Plus) Functions. R package version 1.3-20. **2017**. https://CRAN.R-project.org/package=boot	Bootstraping of confidence intervals for estimates or indices when not already provided in the results
car	3.0.10	Fox, J.; Weisberg, S. *CAR - An R Companion to Applied Regression*; Sage: Thousand Oaks, CA, **2019**.	Data management
CMatching	2.3.0	Cannas, M. CMatching: Matching Algorithms for Causal Inference with Clustered Data. R package version 2.3.0. **2019**. https://CRAN.R-project.org/package=CMatching	Multilevel propensity matching
cocron	1.0.1	Diedenhofen, B. cocron: Statistical Comparisons of Two or more Alpha Coefficients. R package version 1.0.1. **2016**. http://comparingcronbachalphas.org	Comparison of consistency coefficients
effectsize	0.4.0	Ben-Shachar, M.S.; Makowski, D.; Lüdecke, D. Compute and interpret indices of effect size. R package version 0.4.0. **2020**. https://github.com/easystats/effectsize	Computation of effect sizes and their confidence interval
emmeans	1.4.2	Lenth, R. emmeans: Estimated Marginal Means, aka Least-Squares Means. R package version 1.4.2. **2019**. https://CRAN.R-project.org/package=emmeans	Linear Mixed Models
irr	0.84.1	Gamer, M.; Lemon, J.; Fellows, I. irr: Various Coefficients of Interrater Reliability and Agreement. R package version 0.84.1. **2019**. https://CRAN.R-project.org/package=irr	Computation of Intraclass Correlation Coefficients

lme4	1.1.23	Bates, D.; Mächler, M.; Bolker, B. M.; Walker, S. C. Fitting Linear Mixed-Effects Models Using Lme4. *J. Stat. Softw.* **2015**, *67* (1), 1–48. https://doi.org/10.18637/jss.v067.i01.	Linear mixed models
LMERConvenienceFunctions	2.10	Tremblay, A.; Ransijn, J. LMERConvenienceFunctions: A Suite of Functions to Back-Fit Fixed Effects and Forward-Fit Random Effects, as Well as Other Miscellaneous Functions. R Package Version 2.10. **2015**. https://CRAN.R-project.org/package=LMERConvenienceFunctions	Linear Mixed Models
lmerTest	3.0.1	Kuznetsova, A.; Brockhoff, P. B.; Christensen, R. H. B. LmerTest Package: Tests in Linear Mixed Effects Models. *J. Stat. Softw.* **2017**, *82* (13), 1–26. https://doi.org/10.18637/jss.v082.i13.	Linear Mixed Models
lsr	0.5	Navarro, D. J. *Learning Statistics with R: A Tutorial for Psychology Students and Other Beginners*; University of Adelaide: Adelaide, **2015**.	Computation of effect sizes and their confidence interval
MBESS	4.4.3	Kelley, K. CMatching: MBESS: The MBESS R Package. R package version 4.4.3. **2018**. https://CRAN.R-project.org/package=MBESS	Computation of effect sizes and their confidence interval
multtest	2.36.0	Pollard, K. S.; Dudoit, S.; van der Laan, M. J. Multiple Testing Procedures: The Multtest Package and Applications to Genomics. In *Bioinformatics and Computational Biology Solutions Using R and Bioconductor*; Gentleman, R., Carey, V., Huber, W., Irizarry, R., Dudoit, S., Eds.; Springer: Berlin, **2005**; pp 251–272.	*p*-value adjustments for false discovery rate

		https://doi.org/10.1007/0-387-29362-0_15.	
psych	1.8.4	Revelle, W. psych: Procedures for Personality and Psychological Research. R package version 1.8.4. **2018**. https://CRAN.R-project.org/package=psych	Descriptive statistics

Table1. Rating consistency (Intraclass Correlation Coefficients) statistics and results of the comparisons between young and older adults

Item	Young (*n* = 35)	Older adults (*n* = 45)	F	df1	df2	p	adj-p	r
item01-clarity	.82 [.70; .90]	.70 [.54; .81]	1.65	34	44	.120	.303	.13 [-.08; .33]
item02-color	.69 [.50; .83]	.73 [.59; .84]	1.15	44	34	.687	.738	.04 [-.21; .27]
item03-vividness	.87 [.78; .92]	.71 [.56; .82]	2.16	34	44	.017	.113	.20 [.02; .38]
item04-richness of visual details	.75 [.60; .86]	.81 [.71; .88]	1.30	44	34	.437	.575	.07 [-.16; .33]
item05-sound	.79 [.67; .88]	.68 [.51; .80]	1.56	34	44	.162	.368	.12 [-.11; .35]
item06-smell	.74 [.58; .85]	.82 [.73; .89]	1.46	44	34	.256	.419	.10 [-.10; .30]
item07-touch	.81 [.69; .89]	.67 [.51; .80]	1.70	34	44	.097	.303	.14 [-.06; .34]
item08-taste	.83 [.72; .90]	.69 [.52; .81]	1.83	34	44	.059	.246	.16 [-.10; .39]
item09-sensory reliving	.84 [.74; .91]	.67 [.50; .80]	2.04	34	44	.027	.135	.19 [-.02; .40]
item10-auditory reliving	.84 [.74; .91]	.65 [.47; .79]	2.14	34	44	.018	.113	.21 [-.00; .41]
item11-visual reliving	.78 [.64; .87]	.66 [.48; .79]	1.53	34	44	.187	.390	.12 [-.06; .30]
item12-spatial reliving	.79 [.66; .88]	.75 [.62; .85]	1.19	34	44	.577	.687	.05 [-.11; .21]
item13-remember/know	.62 [.38; .79]	.86 [.78; .91]	2.68	44	34	.004	.050	.27 [.03; .49]
item14-formulation in words	.94 [.90; .97]	.81 [.71; .89]	3.07	34	44	.001	.025	.28 [.10; .48]
item15-coherence	.86 [.78; .92]	.77 [.65; .86]	1.67	34	44	.109	.303	.13 [-.06; .32]
item16-confidence in the accuracy of the memory/testify	.78 [.65; .88]	.87 [.80; .92]	1.68	44	34	.121	.303	.13 [-.14; .36]
item17-accessibility	.70 [.51; .83]	.62 [.43; .77]	1.26	34	44	.471	.589	.07 [-.16; .31]
item18-visual perspective/third person	.86 [.77; .92]	.84 [.75; .90]	1.12	34	44	.708	.738	.03 [-.17; .21]
item19-emotional intensity/event	.68 [.48; .82]	.77 [.64; .86]	1.36	44	34	.352	.518	.09 [-.12; .29]
item20-emotional reliving of the event	.69 [.50; .83]	.77 [.65; .86]	1.33	44	34	.391	.543	.08 [-.12; .27]
item21-emotional intensity/memory	.77 [.63; .87]	.78 [.66; .86]	1.02	44	34	.968	.968	.00 [-.20; .24]
item22-self-distancing	.82 [.71; .90]	.74 [.61; .84]	1.42	34	44	.268	.419	.09 [-.14; .30]
item23-rehearsal of memories/talking	.77 [.63; .87]	.66 [.48; .79]	1.48	34	44	.222	.419	.11 [-.14; .32]
item24-personal importance	.79 [.66; .88]	.70 [.54; .81]	1.45	34	44	.244	.419	.10 [-.08; .30]
item25-imagined/real	.81 [.70; .89]	.79 [.68; .87]	1.13	34	44	.705	.738	.03 [-.16; .17]

Note: Bracketed values are the lower and upper limits, respectively, of the 95% confidence interval; F: *F* statistic for the comparison of the ICCs; df1 and df2: numerator and denominator, respectively, degrees of freedom for the *F* statistic; p: raw *p*-value for the *F* statistic; adj-p: *p*-values adjusted for false discovery rate using the Benjamini-Hochberg (1995) procedure; *r*: effect size in *r* metric (thresholds for small, moderate, and large effect size are .10, .30, and .50, respectively, Cohen, 1988)

Table 2. Results of the linear mixed models for predicting APAM scores using age group, gender, age of memory, arousal, positive mood, and specificity of the memory.

Item	Effect	F (df_{num}, df_{den})	Est. (SE)/EMM	p	adj.p	r
item01-clarity	Older adults	32.54 (1, 71.03)	1.43 (0.25)	<.001	<.001	.56 [.38; .70]
	Female	1.01 (1, 84.08)	-0.32 (0.32)	.319	.478	.11 [-.11; .32]
	Age of memory	72.44 (1, 467.39)	-0.81 (0.10)	<.001	<.001	.37 [.29; .44]
	Arousal	10.56 (1, 457.42)	-0.20 (0.06)	.001	.008	.15 [.06; .24]
	Positive Mood	4.87 (1, 461.47)	-0.14 (0.06)	.028	.089	.10 [.01; .19]
	Specificity	0.14 (2, 461.85)		.870	.932	.02 [-.07; .12]
		Global	4.86 (0.30)			
		Multiple	4.91 (0.28)			
		Single	4.84 (0.26)			
	Participant Variance (ICC)	0.82 (.36)				
	Cue Variance (ICC)	0.29 (.13)				
	Residual	1.18				
item02-color	Older adults	0.39 (1, 62.62)	0.12 (0.18)	.532	.688	.08 [-.17; .32]
	Female	1.12 (1, 70.45)	-0.25 (0.24)	.294	.455	.12 [-.11; .35]
	Age of memory	6.42 (1, 442.53)	-0.21 (0.08)	.012	.049	.12 [.03; .21]
	Arousal	5.64 (1, 409.11)	-0.12 (0.05)	.018	.064	.12 [.02; .21]
	Positive Mood	5.92 (1, 417.54)	-0.13 (0.05)	.015	.059	.12 [.02; .21]
	Specificity	0.71 (2, 470.67)		.493	.648	.05 [-.04; .14]
		Global	6.21 (0.22)			
		Multiple	6.15 (0.20)			
		Single	6.05 (0.18)			
	Participant Variance (ICC)	0.39 (.27)				
	Cue Variance (ICC)	0.12 (.08)				
	Residual	0.93				
item03-vividness	Older adults	36.43 (1, 72.01)	1.45 (0.24)	<.001	<.001	.58 [.40; .72]
	Female	0.28 (1, 89.81)	-0.16 (0.30)	.599	.737	.06 [-.15; .26]
	Age of memory	74.98 (1, 470.12)	-0.70 (0.08)	<.001	<.001	.37 [.29; .45]
	Arousal	14.22 (1, 469.53)	-0.20 (0.05)	<.001	.001	.17 [.08; .26]
	Positive Mood	1.77 (1, 472.73)	-0.07 (0.05)	.184	.365	.06 [-.03; .15]
	Specificity	1.25 (2, 452.61)		.287	.454	.07 [-.02; .17]
		Global	4.94 (0.24)			
		Multiple	4.66 (0.22)			
		Single	4.75 (0.20)			
	Participant Variance (ICC)	0.82 (.47)				
	Cue Variance (ICC)	0.11 (.06)				
	Residual	0.81				

(continues)

Item	Effect	F (df_{num}, df_{den})	Est. (SE)/EMM	p	adj.p	r
item04-richness of visual details	Older adults	0.25 (1, 63.89)	0.07 (0.15)	.616	.745	.06 [-.19; .30]
	Female	0.35 (1, 73.75)	-0.11 (0.19)	.559	.713	.07 [-.16; .29]
	Age of memory	16.78 (1, 459.10)	-0.25 (0.06)	<.001	<.001	.19 [.10; .27]
	Arousal	0.00 (1, 437.28)	0.00 (0.04)	.978	.991	.00 [-.09; .10]
	Positive Mood	0.00 (1, 444.32)	0.00 (0.04)	.995	.995	.00 [-.09; .09]
	Specificity	3.68 (2, 467.18)		.026	.086	.12 [.03; .21]
		Global	5.94 (0.16)			
		Multiple	6.08 (0.15)			
		Single	5.84 (0.13)			
	Participant Variance (ICC)	0.26 (.33)				
	Cue Variance (ICC)	0.05 (.07)				
	Residual	0.49				
item05-sound	Older adults	1.46 (1, 68.57)	0.35 (0.29)	.231	.433	.14 [-.10; .37]
	Female	0.29 (1, 77.20)	-0.20 (0.37)	.590	.737	.06 [-.16; .28]
	Age of memory	0.18 (1, 447.41)	-0.05 (0.12)	.675	.787	.02 [-.07; .11]
	Arousal	4.12 (1, 418.70)	0.16 (0.08)	.043	.121	.10 [.00; .19]
	Positive Mood	2.22 (1, 425.99)	0.12 (0.08)	.137	.302	.07 [-.02; .17]
	Specificity	1.58 (2, 469.70)		.207	.398	.08 [-.01; .17]
		Global	4.58 (0.38)			
		Multiple	4.44 (0.35)			
		Single	4.20 (0.33)			
	Participant Variance (ICC)	0.94 (.26)				
	Cue Variance (ICC)	0.49 (.14)				
	Residual	2.15				
item06-smell	Older adults	4.91 (1, 68.63)	0.70 (0.32)	.030	.091	.26 [.02; .47]
	Female	0.01 (1, 81.28)	0.04 (0.40)	.928	.967	.01 [-.21; .23]
	Age of memory	1.94 (1, 466.90)	-0.17 (0.12)	.165	.355	.06 [-.03; .15]
	Arousal	0.20 (1, 456.65)	0.03 (0.08)	.655	.774	.02 [-.07; .11]
	Positive Mood	1.78 (1, 460.40)	-0.11 (0.08)	.183	.365	.06 [-.03; .15]
	Specificity	3.53 (2, 460.64)		.030	.091	.12 [.03; .21]
		Global	3.10 (0.41)			
		Multiple	3.36 (0.38)			
		Single	2.89 (0.36)			
	Participant Variance (ICC)	1.30 (.34)				
	Cue Variance (ICC)	0.60 (.16)				
	Residual	1.89				
item07-touch	Older adults	1.73 (1, 72.27)	0.42 (0.32)	.193	.376	.15 [-.08; .37]
	Female	1.29 (1, 85.68)	-0.47 (0.41)	.259	.436	.12 [-.09; .33]
	Age of memory	4.05 (1, 468.20)	-0.25 (0.12)	.045	.124	.09 [.00; .18]
	Arousal	6.07 (1, 458.60)	0.19 (0.08)	.014	.056	.11 [.02; .20]
	Positive Mood	0.03 (1, 463.14)	0.01 (0.08)	.862	.932	.01 [-.08; .10]
	Specificity	1.30 (2, 462.62)		.272	.449	.07 [-.02; .16]
		Global	3.39 (0.36)			
		Multiple	3.66 (0.33)			
		Single	3.38 (0.30)			
	Participant Variance (ICC)	1.36 (.38)				
	Cue Variance (ICC)	0.32 (.09)				
	Residual	1.94				

(continues)

Item	Effect	F (df_{num}, df_{den})	Est. (SE)/EMM	p	adj.p	r
item08-taste	Older adults	2.61 (1, 71.40)	0.53 (0.33)	.110	.267	.19 [-.05; .40]
	Female	2.29 (1, 85.49)	-0.63 (0.41)	.134	.299	.16 [-.05; .36]
	Age of memory	4.93 (1, 468.05)	-0.27 (0.12)	.027	.088	.10 [.01; .19]
	Arousal	1.29 (1, 461.46)	0.09 (0.08)	.256	.436	.05 [-.04; .14]
	Positive Mood	1.81 (1, 464.07)	0.11 (0.08)	.179	.365	.06 [-.03; .15]
	Specificity	1.24 (2, 457.40)		.292	.455	.07 [-.02; .16]
		Global	2.85 (0.47)			
		Multiple	2.79 (0.45)			
		Single	2.56 (0.43)			
	Participant Variance (ICC)	1.42 (.33)				
	Cue Variance (ICC)	0.97 (.23)				
	Residual	1.87				
item09-sensory reliving	Older adults	10.38 (1, 67.46)	0.91 (0.28)	.002	.011	.37 [.14; .56]
	Female	0.04 (1, 84.95)	-0.07 (0.35)	.837	.923	.02 [-.19; .23]
	Age of memory	33.28 (1, 470.61)	-0.54 (0.09)	<.001	<.001	.26 [.17; .34]
	Arousal	0.61 (1, 470.60)	-0.05 (0.06)	.436	.600	.04 [-.05; .13]
	Positive Mood	0.92 (1, 473.78)	-0.06 (0.06)	.337	.488	.04 [-.05; .13]
	Specificity	3.71 (2, 448.97)		.025	.086	.13 [.04; .22]
		Global	4.69 (0.26)			
		Multiple	4.60 (0.24)			
		Single	4.30 (0.22)			
	Participant Variance (ICC)	1.14 (.49)				
	Cue Variance (ICC)	0.09 (.04)				
	Residual	1.09				
item10-auditory reliving	Older adults	8.10 (1, 70.26)	0.78 (0.27)	.006	.026	.32 [.09; .52]
	Female	1.41 (1, 82.84)	-0.42 (0.35)	.239	.436	.13 [-.09; .34]
	Age of memory	20.64 (1, 467.38)	-0.48 (0.11)	<.001	<.001	.21 [.12; .29]
	Arousal	1.34 (1, 455.72)	-0.08 (0.07)	.247	.436	.05 [-.04; .15]
	Positive Mood	0.00 (1, 460.44)	0.00 (0.07)	.986	.992	.00 [-.09; .09]
	Specificity	1.42 (2, 463.66)		.242	.436	.08 [-.01; .17]
		Global	4.29 (0.30)			
		Multiple	4.25 (0.28)			
		Single	4.03 (0.26)			
	Participant Variance (ICC)	0.98 (.37)				
	Cue Variance (ICC)	0.22 (.08)				
	Residual	1.48				
item11-visual reliving	Older adults	6.45 (1, 73.37)	0.51 (0.20)	.013	.054	.28 [.06; .48]
	Female	1.09 (1, 85.11)	-0.27 (0.26)	.299	.457	.11 [-.10; .32]
	Age of memory	61.31 (1, 463.23)	-0.63 (0.08)	<.001	<.001	.34 [.26; .42]
	Arousal	9.88 (1, 448.13)	-0.16 (0.05)	.002	.010	.15 [.06; .24]
	Positive Mood	1.44 (1, 453.01)	-0.07 (0.05)	.231	.433	.06 [-.04; .15]
	Specificity	1.38 (2, 465.67)		.252	.436	.08 [-.01; .17]
		Global	5.08 (0.25)			
		Multiple	4.94 (0.23)			
		Single	4.83 (0.22)			
	Participant Variance (ICC)	0.51 (.32)				
	Cue Variance (ICC)	0.21 (.13)				
	Residual	0.86				

(continues)

Item	Effect	F (df_{num}, df_{den})	Est. (SE)/EMM	p	adj.p	r
item12-spatial reliving	Older adults	10.26 (1, 72.34)	0.71 (0.22)	.002	.011	.35 [.13; .54]
	Female	1.79 (1, 83.71)	-0.38 (0.29)	.185	.365	.14 [-.07; .35]
	Age of memory	37.22 (1, 462.63)	-0.55 (0.09)	<.001	<.001	.27 [.19; .36]
	Arousal	0.94 (1, 445.83)	-0.06 (0.06)	.332	.488	.05 [-.05; .14]
	Positive Mood	0.02 (1, 451.47)	-0.01 (0.06)	.902	.957	.01 [-.09; .10]
	Specificity	1.33 (2, 467.18)		.266	.443	.08 [-.02; .16]
		Global	4.74 (0.26)			
		Multiple	4.56 (0.23)			
		Single	4.45 (0.22)			
	Participant Variance (ICC)	0.62 (.33)				
	Cue Variance (ICC)	0.16 (.09)				
	Residual	1.09				
item13-remember/know	Older adults	10.09 (1, 72.82)	0.59 (0.19)	.002	.011	.35 [.13; .54]
	Female	0.00 (1, 83.11)	0.01 (0.24)	.965	.985	.00 [-.21; .22]
	Age of memory	12.70 (1, 457.08)	-0.28 (0.08)	<.001	.003	.16 [.07; .25]
	Arousal	9.87 (1, 435.73)	-0.16 (0.05)	.002	.010	.15 [.06; .24]
	Positive Mood	2.38 (1, 442.01)	-0.08 (0.05)	.124	.281	.07 [-.02; .17]
	Specificity	3.52 (2, 468.91)		.030	.091	.12 [.03; .21]
		Global	5.65 (0.23)			
		Multiple	5.91 (0.21)			
		Single	5.60 (0.19)			
	Participant Variance (ICC)	0.41 (.30)				
	Cue Variance (ICC)	0.15 (.11)				
	Residual	0.82				
item14-formulation in words	Older adults	11.00 (1, 74.54)	1.13 (0.34)	.001	.009	.36 [.14; .54]
	Female	0.41 (1, 98.77)	0.27 (0.42)	.522	.681	.06 [-.13; .26]
	Age of memory	2.98 (1, 464.25)	-0.17 (0.10)	.085	.224	.08 [-.01; .17]
	Arousal	1.83 (1, 467.52)	-0.09 (0.06)	.177	.365	.06 [-.03; .15]
	Positive Mood	0.03 (1, 470.18)	-0.01 (0.07)	.855	.932	.01 [-.08; .10]
	Specificity	0.10 (2, 441.47)		.908	.957	.02 [-.07; .11]
		Global	3.59 (0.30)			
		Multiple	3.57 (0.27)			
		Single	3.52 (0.26)			
	Participant Variance (ICC)	1.76 (.58)				
	Cue Variance (ICC)	0.11 (.03)				
	Residual	1.19				
item15-coherence	Older adults	32.38 (1, 67.11)	1.48 (0.26)	<.001	<.001	.57 [.38; .71]
	Female	1.96 (1, 82.78)	0.46 (0.33)	.166	.355	.15 [-.07; .36]
	Age of memory	52.11 (1, 472.03)	-0.65 (0.09)	<.001	<.001	.32 [.23; .39]
	Arousal	4.51 (1, 468.99)	-0.12 (0.06)	.034	.101	.10 [.01; .19]
	Positive Mood	0.28 (1, 472.55)	-0.03 (0.06)	.596	.737	.02 [-.07; .11]
	Specificity	6.80 (2, 452.18)		.001	.008	.17 [.08; .26]
		Global	4.01 (0.25)a,b			
		Multiple	3.73 (0.22)a			
		Single	4.21 (0.20)b			
	Participant Variance (ICC)	0.94 (.46)				
	Cue Variance (ICC)	0.08 (.04)				
	Residual	1.03				

(continues)

Item	Effect	F (df_{num}, df_{den})	Est. (SE)/EMM	p	adj.p	r
item16-confidence in the accuracy of the memory/testify	Older adults	9.67 (1, 67.96)	0.76 (0.24)	.003	.013	.35 [.12; .55]
	Female	0.03 (1, 84.02)	0.05 (0.31)	.867	.932	.02 [-.20; .23]
	Age of memory	53.17 (1, 470.82)	-0.62 (0.08)	<.001	<.001	.32 [.23; .40]
	Arousal	0.22 (1, 468.56)	0.03 (0.05)	.636	.763	.02 [-.07; .11]
	Positive Mood	0.00 (1, 472.28)	0.00 (0.06)	.957	.983	.00 [-.09; .09]
	Specificity	0.58 (2, 453.51)		.561	.713	.05 [-.04; .14]
		Global	5.12 (0.24)			
		Multiple	5.30 (0.22)			
		Single	5.19 (0.20)			
	Participant Variance (ICC)	0.84 (.46)				
	Cue Variance (ICC)	0.10 (.06)				
	Residual	0.90				
item17-accessibility	Older adults	0.12 (1, 65.70)	0.08 (0.25)	.733	.846	.04 [-.20; .28]
	Female	0.07 (1, 76.43)	-0.08 (0.32)	.795	.883	.03 [-.20; .25]
	Age of memory	0.31 (1, 462.93)	-0.05 (0.10)	.580	.731	.03 [-.07; .12]
	Arousal	0.63 (1, 444.88)	-0.05 (0.06)	.429	.596	.04 [-.06; .13]
	Positive Mood	2.40 (1, 451.17)	-0.10 (0.07)	.122	.281	.07 [-.02; .16]
	Specificity	9.33 (2, 465.90)		<.001	.001	.20 [.11; .28]
		Global	5.63 (0.27)b			
		Multiple	5.62 (0.24)b			
		Single	5.06 (0.22)a			
	Participant Variance (ICC)	0.76 (.34)				
	Cue Variance (ICC)	0.15 (.07)				
	Residual	1.31				
item18-visual perspective/third person	Older adults	1.32 (1, 72.13)	0.42 (0.36)	.254	.436	.13 [-.10; .35]
	Female	0.91 (1, 90.14)	0.44 (0.46)	.342	.488	.10 [-.11; .30]
	Age of memory	19.12 (1, 472.55)	0.53 (0.12)	<.001	<.001	.20 [.11; .28]
	Arousal	3.67 (1, 471.42)	0.15 (0.08)	.056	.153	.09 [-.00; .18]
	Positive Mood	2.80 (1, 473.00)	-0.14 (0.08)	.095	.242	.08 [-.01; .17]
	Specificity	0.79 (2, 440.28)		.455	.615	.06 [-.03; .15]
		Global	3.64 (0.32)			
		Multiple	3.56 (0.29)			
		Single	3.78 (0.27)			
	Participant Variance (ICC)	1.87 (.49)				
	Cue Variance (ICC)	0.08 (.02)				
	Residual	1.85				
item19-emotional intensity/event	Older adults	4.46 (1, 71.44)	0.40 (0.19)	.038	.110	.24 [.01; .45]
	Female	0.01 (1, 81.80)	-0.03 (0.25)	.912	.957	.01 [-.21; .23]
	Age of memory	1.14 (1, 457.98)	0.08 (0.08)	.285	.454	.05 [-.04; .14]
	Arousal	1.01 (1, 437.23)	0.05 (0.05)	.315	.477	.05 [-.05; .14]
	Positive Mood	0.09 (1, 443.51)	-0.02 (0.05)	.767	.875	.01 [-.08; .11]
	Specificity	4.62 (2, 468.45)		.010	.045	.14 [.05; .23]
		Global	5.16 (0.23)a			
		Multiple	5.61 (0.21)b			
		Single	5.31 (0.20)a			
	Participant Variance (ICC)	0.44 (.31)				
	Cue Variance (ICC)	0.15 (.11)				
	Residual	0.85				

(continues)

Item	Effect	F (df_{num}, df_{den})	Est. (SE)/EMM	p	adj.p	r
item20-emotional reliving of the event	Older adults	9.70 (1, 68.01)	0.76 (0.24)	.003	.013	.35 [.13; .55]
	Female	0.59 (1, 81.05)	0.24 (0.31)	.444	.605	.09 [-.14; .30]
	Age of memory	37.53 (1, 469.35)	-0.56 (0.09)	<.001	<.001	.27 [.19; .35]
	Arousal	1.82 (1, 459.48)	0.08 (0.06)	.178	.365	.06 [-.03; .15]
	Positive Mood	0.00 (1, 464.30)	0.00 (0.06)	.957	.983	.00 [-.09; .09]
	Specificity	3.69 (2, 461.27)		.026	.086	.13 [.03; .21]
		Global	4.54 (0.25)			
		Multiple	4.67 (0.23)			
		Single	4.31 (0.21)			
	Participant Variance (ICC)	0.78 (.39)				
	Cue Variance (ICC)	0.12 (.06)				
	Residual	1.10				
item21-emotional intensity/memory	Older adults	1.39 (1, 68.12)	0.30 (0.25)	.242	.436	.14 [-.10; .37]
	Female	2.79 (1, 82.26)	0.54 (0.32)	.099	.247	.18 [-.04; .38]
	Age of memory	2.70 (1, 469.30)	-0.15 (0.09)	.101	.248	.08 [-.01; .17]
	Arousal	0.09 (1, 463.48)	0.02 (0.06)	.770	.875	.01 [-.08; .10]
	Positive Mood	0.25 (1, 466.72)	-0.03 (0.06)	.614	.745	.02 [-.07; .11]
	Specificity	8.23 (2, 457.22)		<.001	.002	.19 [.10; .27]
		Global	4.63 (0.30)b			
		Multiple	4.57 (0.28)b			
		Single	4.10 (0.26)a			
	Participant Variance (ICC)	0.88 (.39)				
	Cue Variance (ICC)	0.29 (.13)				
	Residual	1.10				
item22-self-distancing	Older adults	5.95 (1, 76.17)	-0.79 (0.32)	.017	.062	.27 [.05; .47]
	Female	0.08 (1, 94.29)	0.12 (0.41)	.780	.880	.03 [-.17; .23]
	Age of memory	105.85 (1, 472.98)	1.17 (0.11)	<.001	<.001	.43 [.35; .50]
	Arousal	2.84 (1, 472.00)	0.12 (0.07)	.093	.240	.08 [-.01; .17]
	Positive Mood	0.66 (1, 448.05)	-0.06 (0.08)	.417	.584	.04 [-.05; .13]
	Specificity	6.00 (2, 363.86)		.003	.013	.18 [.08; .28]
		Global	5.06 (0.28)b			
		Multiple	4.88 (0.25)b			
		Single	4.45 (0.23)a			
	Participant Variance (ICC)	1.48 (.47)				
	Cue Variance (ICC)	0.01 (.00)				
	Residual	1.67				
item23-rehearsal of memories/talking	Older adults	0.53 (1, 63.12)	0.20 (0.27)	.469	.622	.09 [-.16; .33]
	Female	1.16 (1, 70.99)	-0.38 (0.35)	.285	.454	.13 [-.11; .35]
	Age of memory	0.07 (1, 442.78)	0.03 (0.12)	.788	.882	.01 [-.08; .11]
	Arousal	0.21 (1, 409.99)	0.04 (0.08)	.645	.768	.02 [-.07; .12]
	Positive Mood	0.91 (1, 418.40)	-0.08 (0.08)	.341	.488	.05 [-.05; .14]
	Specificity	1.25 (2, 470.34)		.287	.454	.07 [-.02; .16]
		Global	3.43 (0.34)			
		Multiple	3.65 (0.31)			
		Single	3.37 (0.29)			
	Participant Variance (ICC)	0.84 (.27)				
	Cue Variance (ICC)	0.33 (.10)				
	Residual	2.00				

(continues)

Item	Effect	F (df_{num}, df_{den})	Est. (SE)/EMM	p	adj.p	r
item24-personal importance	Older adults	2.49 (1, 69.21)	0.45 (0.28)	.119	.278	.19 [-.05; .40]
	Female	2.49 (1, 80.16)	0.57 (0.36)	.119	.278	.17 [-.05; .38]
	Age of memory	0.55 (1, 461.41)	0.08 (0.11)	.459	.615	.03 [-.06; .13]
	Arousal	0.84 (1, 444.35)	0.07 (0.07)	.359	.509	.04 [-.05; .14]
	Positive Mood	0.95 (1, 449.42)	-0.08 (0.08)	.330	.488	.05 [-.05; .14]
	Specificity	1.36 (2, 465.23)		.259	.436	.08 [-.01; .17]
		Global	3.57 (0.38)			
		Multiple	3.77 (0.35)			
		Single	3.49 (0.33)			
	Participant Variance (ICC)	1.00 (.31)				
	Cue Variance (ICC)	0.51 (.16)				
	Residual	1.74				
item25-imagined/real	Older adults	17.54 (1, 73.80)	0.56 (0.13)	<.001	.001	.44 [.23; .61]
	Female	0.17 (1, 86.55)	0.07 (0.17)	.677	.787	.04 [-.17; .25]
	Age of memory	17.69 (1, 467.92)	-0.22 (0.05)	<.001	<.001	.19 [.10; .28]
	Arousal	5.78 (1, 453.37)	-0.08 (0.03)	.017	.062	.11 [.02; .20]
	Positive Mood	3.18 (1, 452.26)	-0.06 (0.04)	.075	.201	.08 [-.01; .17]
	Specificity	4.42 (2, 435.68)		.013	.052	.14 [.05; .23]
		Global	6.30 (0.13)			
		Multiple	6.11 (0.11)			
		Single	6.33 (0.10)			
	Participant Variance (ICC)	0.22 (.37)				
	Cue Variance (ICC)	0.01 (.02)				
	Residual	0.37				

Note: F: parameter estimate; df_{num}: numerator degrees of freedom; df_{den}: denominator degrees of freedom; Est. (SE)/EMM: parameter estimate and its standard error; p: p-value; adj.p.: p-value adjusted for false discovery rate (Benjamini & Hochberg, 1995); *r*: effect size and its 95% confidence interval; Global: estimated marginal mean (EMM) and standard error for item 26-specificity category: event that extended for a period greater than on day; Multiple: EMM and standard error for item 26-specificity category: summary or merging of similar events; Single: EMM and standard error for item 26-specificity category: event that occurred once within a single day and place; letters beside EEMs indicate significant differences according to the compact letter display system (p-value adjustment: Tukey method for comparing a family of 3 estimates); ICC: intraclass correlation coefficient for the random effect.

Appendix A. Assessment of the Phenomenology of Autobiographical Memory (APAM)

- Item01: My memory for this event is 1= dim; 7= sharp/clear
- Item02: My memory for this event is 1= black and white; 7= entirely colored
- Item03: My memory for this event is 1= vague; 7 = very vivid
- Item04: My memory for this event involves visual detail 1 = little or none; 7 = a lot
- Item05: My memory for this event involves sound 1 = little or none; 7 = a lot
- Item06: My memory for this event involves smell 1 = little or none; 7 = a lot
- Item07: My memory for this event involves touch 1 = little or none; 7 = a lot
- Item08: My memory for this event involves taste 1 = little or none; 7 = a lot
- Item09: As I remember the event, I feel as though I am *reliving* the original event (1= not at all ; 7 = as clearly as if it were happening right now)
- Item10: As I remember the event, I can hear it in my mind (1= not at all ; 7 = as clearly as if it were happening right now)
- Item11: As I remember the event, I can see it in my mind (1= not at all ; 7 = as clearly as if it were happening right now)
- Item12: As I remember the event, I can recall the setting where it occurred (1= not at all ; 7 = as clearly as if it were happening right now)
- Item13: Sometimes people know something happened to them without being able to actually remember it. As I think about the event, I can actually remember it rather than just knowing that it happened (1= completely disagree; 7= completely agree)
- Item14: As I remember the event, it comes to me in words (1= completely disagree; 7= completely agree)
- Item15: As I remember the event, it comes to me in words or in pictures as a coherent story or episode and not as an isolated fact, observation, or scene (1= completely disagree; 7= completely agree)

- Item16: You are confident enough in your memory of the event to testify in a court of law (1= completely disagree; 7= completely agree)
- Item17: This memory just sprang to my mind when I read the instructions (1= completely disagree; 7= completely agree)
- Item18: I view this memory as if I was an observer to the experience (1= completely disagree; 7= completely agree)
- Item19: My feelings at the time of the event were intense (1= not at all; 7 = a lot)
- Item20: As I remember the event, I can feel now the emotions that I felt then (1= not at all ; 7 = as clearly as if it were happening right now)
- Item21. As I remember the event, my feelings are intense (1= not at all; 7 = a lot)
- Item22: I feel like the person in this memory is a different person than who I am today (1= completely disagree; 7= completely agree)
- Item23: Since it happened, I have talked about this event (1= never; 7= very often)
- Item24: This memory is significant for my life because it imparts an important message for me or represents an anchor, critical juncture, or a turning point (1= completely disagree; 7= completely agree)
- Item25: I believe the event in my memory really occurred in the way I remember it and that I have not imagined or fabricated anything that did not occur. (1 = 100% imaginary; 7 = 100% real)
- Item26:To the best of your knowledge, is the memory of :
    - ❑ an event that occurred once at one particular time and place
    - ❑ a summary or merging of many similar or related events
    - ❑ events that occurred over a fairly continuous extended period of time lasting more than a day
- Item27: When did the event you recalled take place?
    - ❑ In the last week

- ❑ In the last month
- ❑ In the last six months
- ❑ In the last year
- ❑ 2-5 years ago
- ❑ 6-10 years ago
- ❑ 11-15 years ago
- ❑ 16-30 years ago
- ❑ 31-50 years ago
- ❑ more than 50 years ago
